# Electrochemical Magnetic Immunoassay for the Determination of the Fish Allergen β-Parvalbumin

**DOI:** 10.3390/bios14120639

**Published:** 2024-12-23

**Authors:** José Pedro Rocha, Maria Freitas, Dulce Geraldo, Fátima Bento, Cristina Delerue-Matos, Henri P. A. Nouws

**Affiliations:** 1 REQUIMTE/LAQV, Instituto Superior de Engenharia do Porto, Instituto Politécnico do Porto, Rua Dr. António Bernardino de Almeida 431, 4249-015 Porto, Portugal; jose.rocha@graq.isep.ipp.pt (J.P.R.); cmm@isep.ipp.pt (C.D.-M.); 2 Centro de Química, Campus de Gualtar, Universidade do Minho, 4710-057 Braga, Portugal; gdulce@quimica.uminho.pt (D.G.); fbento@quimica.uminho.pt (F.B.)

**Keywords:** electrochemical biosensor, β-parvalbumin, fish allergen, maleimide-modified magnetic-beads, immunomagnetic assay, chronoamperometry, food safety, seafood

## Abstract

β-parvalbumin (β-PV) is the primary fish allergen responsible for most allergic reactions in individuals sensitive to fish. To ensure food safety, a sandwich-based magnetic immunoassay was developed using maleimide-functionalized magnetic beads (NH-MBs). Specific anti-β-PV antibodies were immobilized on these MBs, and a screen-printed carbon electrode was employed as the electrochemical transducer. A linear concentration range from 10 to 1000 ng/mL, a limit of detection of 1.8 ng/mL, and a limit of quantification of 7.1 ng/mL were achieved. Nineteen commercial food samples were analyzed to assess the potential of the sensor for routine use in food quality control. Important factors such as protein source and food processing (e.g., boiling, grilling, and frying) and preservation (e.g., in oil, and vacuum) were evaluated. The validated results confer the usefulness of the assay for food quality control.

## 1. Introduction

Parvalbumins are a class of proteins belonging to the calcium-binding protein group, with β-parvalbumin (β-PV) and α-parvalbumin (α-PV) standing out as the two main isoforms [1]. These proteins are present in vertebrates and can be found in higher concentrations in muscle tissues. Their distribution in fish is heterogeneous, with a higher likelihood of being present in bony fish species, the main marine species consumed globally [2,3]. β-PV is a fish allergen and is particularly interesting for analysis since it is responsible for most allergic reactions in hypersensitized individuals to seafood [4]. It triggers a cascade of allergic reactions, with symptoms ranging from mild (e.g., hives and swelling) to severe, including anaphylaxis [5]. Therefore, the European Union’s legislation included fish in the mandatory labeling requirements for pre-packaged commercial products (Regulation EU, No 1169/2011). However, the law has not been sufficient to prevent the risks and accidents associated with seafood consumption, threatening the health and lives of individuals with fish allergies. As a result, 5 mg of β-PV was recently declared by the World Health Organization as the reference dose (RfD) for fish and fishery products, corresponding to the total protein content derived from the allergenic source that does not cause significant side effects [6].

Detecting and monitoring β-PV in the food industry to improve product label information is crucial, as it prevents adverse reactions and ensures compliance with food safety standards, thereby protecting consumers from fish allergies [7]. In countries with high fish consumption, such as Japan, Norway, and Portugal, detecting β-PV is of utmost importance [8]. In this context, analytical methodologies have been applied to ensure the food safety of seafood products, namely Enzyme-Linked Immunosorbent Assay (ELISA) [9,10], Polymerase Chain Reaction (PCR) [11], liquid chromatography–mass spectrometry (LC-MS) [7], capillary electrophoresis (CE) [12], and Surface Plasmon Resonance (SPR) [13]. Despite their inherent advantages, including highly sensitive analysis, these techniques involve costly instrumentation, are difficult to employ in routine analysis, have long analysis times, and require specialized analysts. For this reason, the upcoming approaches for rapid analysis emphasize advantages, namely the portability of the equipment, leading to the development of simpler methodologies for a feasible and cost-effective analysis [14].

The portability, low cost, rapid response time, and capability for in situ analysis are key advantages that highlight the potential of biosensors to be implemented in food quality control [15]. Electrochemical biosensors represent an innovative approach to rapidly and efficiently detect proteins, such as allergens [16,17,18,19,20,21]. Recent advances in biosensing have enabled the highly selective determination of β-PV [22].

Currently, the immobilization of antibodies on magnetic beads (MBs) is increasingly applied for the construction of immunoassays, as this promising approach offers several advantages [23,24,25]. The ease of handling is a main benefit since the micro- or nanoparticles can be separated from complex solutions by applying an external magnetic field, allowing efficient washing and reducing background interference [26]. Another significant advantage is the high sensitivity, since these materials are porous and possess a large surface area. Consequently, antibodies interaction increases, resulting in low analytical thresholds [27]. The flexibility of functionalization is advantageous as the bead’s core (e.g., iron oxide) can easily be coated with materials containing functional groups (e.g., amines, carboxylic acids, or thiols), which interact specifically with the antibody’s functional groups without compromising their chemical integrity [28]. This enables a greener and simpler methodology to develop magnetic immunoassays.

In this work, maleimide-functionalized MBs (NH-MBs) were used to immobilize the bioreceptor (β-PV antibody) through a chemical bond established via the reactivity of the amine functional groups on the MBs and the thiol groups on the antibody by the Michael addition reaction. A sandwich magnetic immunoassay was constructed to develop a feasible electrochemical immunoassay, applied to the analysis of commercial food samples, including fish and fish-derived products that contain, may contain, or do not contain β-PV. The electrochemical signal was obtained by chronoamperometry during an enzymatic reaction (HRP/TMB-H_2_O_2_) conducted on a miniaturized electrochemical transducer (screen-printed carbon electrode, SPCE), containing the magnetic sandwich complex on its surface. Chronoamperometric analysis using HRP/TMB-H_2_O_2_ can be performed by applying a potential of 0 V, where the oxidized form of TMB is electrochemically reduced [18,22]. This low potential minimizes the likelihood of other species undergoing oxidation or reduction processes, increasing the selectivity of the assay. The results were compared with a conventional ELISA to assess the assay’s accuracy.

## 2. Materials and Methods

### 2.1. Equipment, Reagents, and Solutions

Electrochemical measurements (chronoamperometry) were carried out with a potentiostat/galvanostat (PGSTAT101, Metrohm Autolab, Utrecht, The Netherlands). A connector (DRP-CAC, Metrohm DropSens, Oviedo, Spain) was used to interface this equipment with the transducer. The transducer consisted of a miniaturized electrochemical cell; an SPCE (DRP110, Metrohm DropSens, Oviedo, Spain), composed of a carbon-based working electrode (WE, d = 4 mm); a carbon-based auxiliary electrode (AE); and a silver-based pseudo-reference electrode (RE). Scanning electron microscopy (SEM) and energy-dispersive spectroscopy (EDS) were performed using a FEI Quanta 400FEG ESEM/EDAX Genesis X4 M equipment (Hillsboro, OR, USA), with a detector type SUTW Sapphire analysis system of resolution 132.19. The SEM analyses were conducted at the “Centro de Materiais da Universidade do Porto (CEMUP)”. A HulaMixerTM Sample Mixer (Invitrogen–Thermo Fisher Scientific, Oslo, Norway) was used in the magnetic assay. For the extraction of β-PV from the selected foods, a block thermostat (Tembloc, P-Selecta, Barcelona, Spain), a Heraeus Megafuge 16R centrifuge (Thermo Fisher Scientific, Osterode am Harz, Germany), and a FrescoTM microcentrifuge (Heraeus-Fresco 21, Thermo Fisher Scientific, Osterode am Harz, Germany) were used. A multi-mode microplate reader (Synergy HT W/TRF) and Gen5 software (v2.0, BioTek Instruments, Winooski, VT, USA) were used to validate the immunosensor’s results using an ELISA Kit.

Maleimide Hi-sur Mag magnetic beads (NH-MBs, 10 mg, d = 1 µm) were purchased from RayBiotech (Peachtree Corners, GA, USA). The capture antibody (unconjugated polyclonal β-PV antibody, CAb), detection antibody (HRP-conjugated β-PV polyclonal antibody, DAb-HRP), fish allergen (recombinant *Gadus morhua* subsp. callarias Parvalbumin, β-PV), and ELISA kit were acquired from MyBioSource. 3,3’,5,5’-Tetramethylbenzidine liquid substrate (TMB-H_2_O_2_ K-Blue reagent), bovine serum albumin (BSA), potassium hydrogen phosphate, potassium dihydrogen phosphate, sodium carbonate, and Tween^®^20 were obtained from Merck (Darmstadt, Germany). Sodium chloride was purchased from VWR Chemicals (Porto, Portugal). Phosphate-buffered saline (pH 7.4, 10 mM PBS with 5 mM EDTA and 0.01% Tween^®^20) was used throughout the work (PBST). Other buffers used throughout the work included B1 (PBST with BSA 0.6% (*w*/*v*)) and B2 (PBST with BSA 0.3% (*w*/*v*)). An extraction buffer (16 mM Na_2_CO_3_ containing 128 mM NaCl, pH 9.6) was used for sample preparation.

### 2.2. Electrochemical Assay

**Figure 1:**
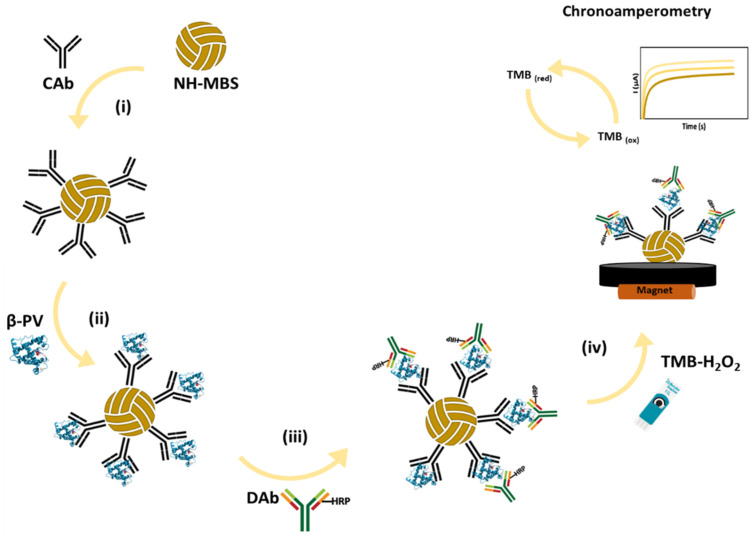
Schematic representation of the magnetic sandwich immunoassay: (i) NH-MB biomodification with CAb, and addition of (ii) β-PV, (iii) DAb-HRP, and (iv) TMB-H₂O₂, with exemplification of the analytical signal recorded by chronoamperometry.

The magnetic assay was carried out in microtubes, and a DynaMag^TM^-2 magnetic rack (Life Technologies, Oslo, Norway) was employed for separation. Washing steps were performed between each biomolecule incubation step (2× with PBST, 2 min, under gentle agitation). An 8 µL aliquot of NH-MBs (per electrode) was used to perform the immunoassay. Figure 1 presents a schematic representation of the developed sensor.

The magnetic sandwich-type immunoassay was performed as follows: (i) the CAb (100 µL, 2 µg/mL) was added to the NH-MBs suspension (overnight, under gentle agitation, RT) and the bioreceptor covalently bonded to the NH-MBs through the Michael addition reaction. The immunoassay consisted of (ii) incubation of the allergen or sample (β-PV in B1, 100 µL, 30 min, under gentle agitation), followed by the addition of (iii) DAb-HRP (dilution of 165× in B2, 100 µL, 30 min, under gentle agitation). Afterward, the NH-MBs were washed, and 8 µL of the suspension was placed on the WE surface, with a magnet (d = 4 mm) placed below it. The excess solution was carefully removed using a micropipette, ensuring that the magnetic beads were not dislodged from the transducer surface, and (iv) a 40 µL aliquot of TMB-H_2_O_2_ was placed on the SPCE, covering the 3-electrode system. The amperograms were recorded at 0.0 V (corresponding to ~0.12 V vs. the standard hydrogen electrode) for 60 s. For analytical purposes, the (absolute) average currents measured during the last 10 s (50–60 s), the time interval in which the current is stable, were used. All experiments were performed in triplicate, at room temperature (25 °C).

### 2.3. Sample Preparation

Food products were purchased in local supermarkets (Porto, Portugal). A sample (1 g) of each product was ground and placed in a glass vial, and 10 mL of the extraction buffer was added. The mixture was then heated under gentle agitation (60 °C, 30 min). Subsequently, the mixture was centrifuged (10 min, 5000 rpm). After centrifugation, 1 mL of the supernatant was collected in a microtube and further centrifuged (3 min, 10,000 rpm). Sample extracts were diluted (100, 300 or 400×) and stored at −20 °C until analysis.

### 2.4. ELISA Procedure

A 96-well polystyrene microtiter plate (Thermo Scientific, Osterode am Harz, Germany) was coated with CAb (0.5 μg/mL in 0.04 M hydrogen carbonate buffer, pH 9.6, 100 μL/well, overnight at 4–8 °C). The plate was allowed to reach room temperature (RT), washed 3× with phosphate-buffered saline (PBS, 10 mM, containing 137 mM NaCl, pH 7.4), and the wells were blocked with 0.5% (*w*/*v*) BSA in PBS pH 7.4 (100 μL/well, 30 min, RT). The plate was washed 3× with 0.1 M PBS, pH 7.4, containing 0.05% Tween 20. Samples were diluted (FD: 100×, 300× and 400×, depending on the sample) with 0.1 M PBS containing BSA 0.1% (100 μL/well) and incubated for 1 h at RT, under gentle agitation. After a washing step, the DAb-HRP was added (100 μL/well, FD: 600×, 1 h, RT), and β-PV was quantified after the addition of K-Blue TMB-substrate (100 μL/well, 20 min, RT). The reaction was stopped by adding 0.5 M H2SO4 (25 μL/well), and the absorbance was measured at 450 nm on the microplate reader after 5 min. Each sample was analyzed in triplicate.

## 3. Results

### 3.1. Immunoassay Optimization

**Figure 2:**
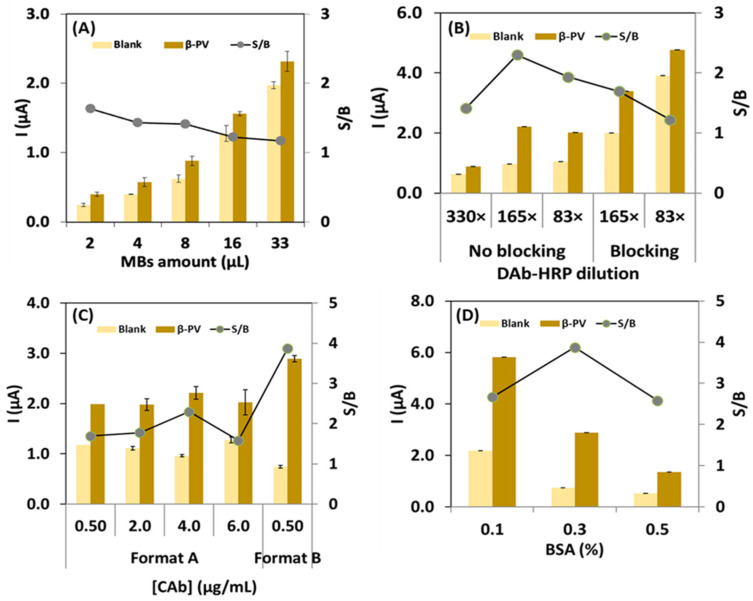
Immunosensor optimization in absence (B, 0 ng/mL) and presence of β-PV (S, 2000 ng/mL), and the corresponding S/B ratio: (**A**) effect of the amount of NH-MB suspension; (**B**) blocking with or without BSA before β-PV and DAb-HRP incubation; (**C**) concentration of CAb (µg/mL) and assay format (A, simultaneous incubation of β-PV and DAb-HRP; and B, step-by-step assay); and (**D**) amount of BSA added to the DAb-HRP solution.

The experimental parameters/variables of the assay were optimized to achieve the highest S/B ratio between the sensor’s response in the presence (2000 ng/mL, signal, S) and absence (blank, B) of β-PV. The amount of NH-MBs deposited on the WE, BSA as blocking agent, DAb-HRP dilution with and without a blocking agent (BSA), CAb concentration, and assay format were studied, and the results are presented in Figure 2.

In this work, NH-MBs were used, so the antibody immobilization occurred through the thiol-Michael addition reaction, binding between a carbon in the maleimide ring and the SH groups of the CAb. Briefly, the Michael addition reaction is characterized by a nucleophilic addition that occurs when the maleimide group is in contact with the SH groups of the antibody. The sulfur from the SH group, which is highly electronegative (nucleophilic), attacks the electrophilic carbon in the five-membered ring with an unsaturated imide group. The chemical reaction is efficient under neutral or slightly alkaline conditions (pH between 6.5 and 7.5), without additional catalysts, allowing for a rapid and selective conjugation within a short time frame (around 2 h), depending on the reagent’s concentration. Thus, the CAb is attached to the surface of the NH-MBs through a specific orientation that allows efficient binding to β-PV.

To optimize the amount of NH-MBs, several volumes of the NH-MBs suspension were tested (2, 4, 8, 16, and 33 µL per electrode), and the results depicted in Figure 2A demonstrate that the S/B ratio varied between 1.6 for 2 μL and 1.2 for 33 μL. This result indicated that the sensor response tended to decrease with the increase in MB amount, especially when it exceeded 8 μL. Thus, with a larger volume of beads (8 µL), the impedance related to the electron transfer during the signal transduction process is expected to be higher. Although 8 μL per electrode did not correspond to the highest S/B ratio or the maximum response of the sensor, this experimental parameter was chosen because, below this amount, it becomes difficult to visually observe the spheres, requiring a longer time for magnetic attraction, and leads to a longer assay time.

Subsequently, the combined effect of blocking with BSA (15 min, prior to β-PV incubation) and DAb-HRP dilution (83×, 165×, and 330×) was tested (Figure 2B). In the absence of the blocking step, the highest S/B ratio (2.3) was achieved for a DAb-HRP dilution of 165×. In contrast, with the blocking step, for the same dilution, a lower S/B value was observed (1.7). Thus, a DAb-HRP dilution of 165×, without prior blocking, was chosen to proceed with the optimization of the sensor.

Then, the CAb concentration was optimized, and two distinct formats were explored: Format A, corresponding to the simultaneous incubation of β-PV and DAb-HRP; and Format B, corresponding to a “step-by-step” approach, where β-PV and DAb-HRP were incubated sequentially. As can be observed in Figure 2C, in Format A, the variation in CAb concentration (0.50, 2.0, 4.0, and 6.0 μg/mL) had a minimal impact on the sensor response, with low S/B ratios (ranging from 1.6 to 2.3). For Format B, 0.50 μg/mL was selected because it provided better precision, and less antibodies were needed to perform the assay. With this format, the highest S/B ratio (3.9) was achieved. This is probably because a step-by-step assay allows for a more efficient binding, since intermediate washing steps remove unbound biomolecules and avoid excessive competition.

After the assessment of these parameters, an additional study involving the addition of BSA (0.1, 0.3, or 0.5% (*w*/*v*)) to the DAb-HRP solution (2.0 µg/mL) was performed (Figure 2D). The highest response was observed for BSA 0.3% (*w*/*v*), resulting in an S/B ratio of 3.9. At 0.1% (*w*/*v*), the DAb-HRP reactivity is significantly high, leading to non-specific interactions, thus increasing the blank signal and decreasing the S/B ratio to 2.7. On the other hand, at a higher BSA concentration (0.5% (*w*/*v*)), the DAb-HRP reactivity is lower because the non-specific reactive groups are blocked by BSA, reducing the blank signal. However, under these conditions, a low sensor response to the presence of β-PV is observed, with a decreased S/B ratio to 2.6. Therefore, the optimal result was found using BSA 0.3% (*w*/*v*) in the DAb-HRP solution.

Moreover, the sensor’s non-specific interactions were assessed in the absence of (i) CAb, (ii) β-PV, (iii) DAb-HRP, and (iv) TMB, and compared to (v) an assay in the presence of all the biomolecules from the optimized assay. Although in the absence of CAb an analytical signal was obtained, the sensitivity was much lower compared to the presence of all biomolecules that produce efficient detection. It was observed that the absence of other biomolecules (ii) to (iv) did not yield a significant analytical signal, showing their impact on the construction of the immunoassay.

### 3.2. Transducer Surface Characterization

**Figure 3:**
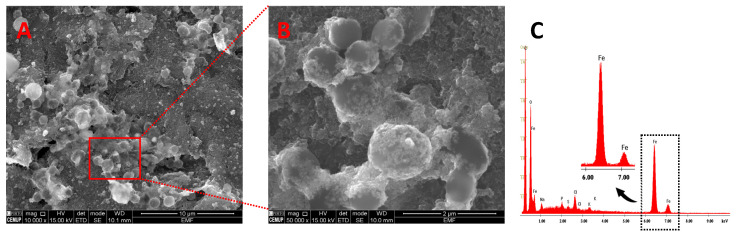
Electrode surface characterization: (**A**,**B**) scanning electron microscopy micrographs (scale bar: 10 and 2 µm, respectively; magnification: 10,000 and 50,000×, respectively) and (**C**) energy-dispersive X-ray spectroscopy spectra of the transducer surface containing the NH-MBs.

For the electrode surface characterization, containing the NH-MBs, SEM and EDS were carried out. In Figure 3A,B, the SEM micrographs show spherical particles with an average size of 1 µm (as specified by the manufacturer) that were uniformly distributed on the WE surface without agglomerations, enhancing the electrochemical signal. Additionally, the EDS analysis indicates the presence of iron, present in the magnetic core of the NH-MBs (Figure 3C).

### 3.3. Analytical Characteristics and Assay Performance

**Figure 4:**
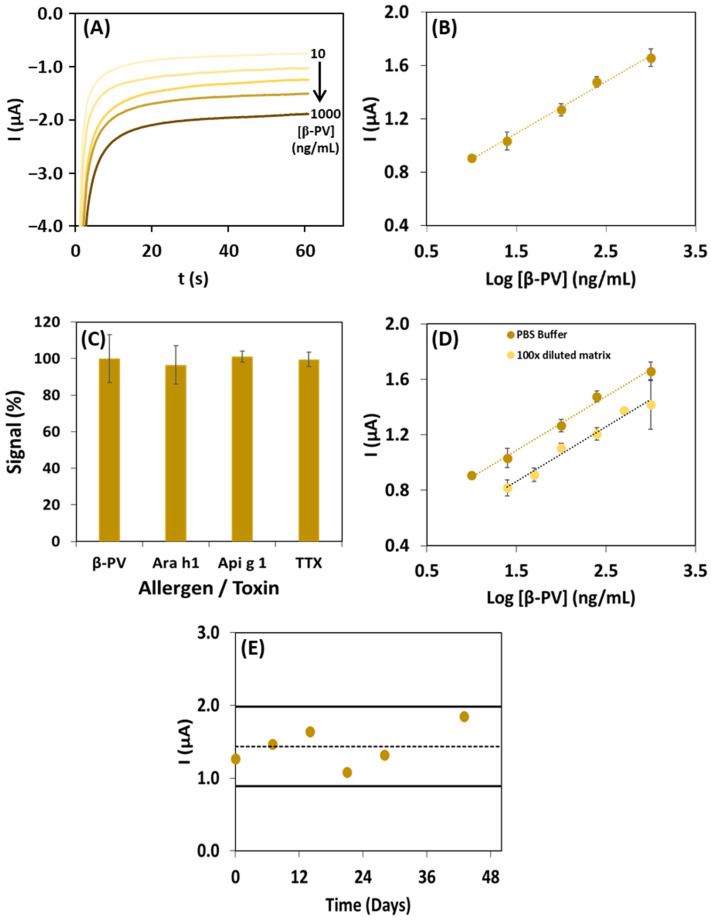
Analytical characterization of the immunoassay: (**A**) chronoamperograms of increasing concentrations of β-PV (10, 25, 100, 250, and 1000 ng/mL); (**B**) calibration curve obtained for the immunoassay; (**C**) interference study in the presence of different possible interferents (Ara h 1, Api g 1, and TTX) at concentrations of 1000 ng/mL; (**D**) comparison of curves of β-PV in buffer and a food extract; and (**E**) stability study over 43 days (dashed line, average of the measurements; solid lines, average ± 2 s (s = standard deviation).

Representative chronoamperograms of β-PV standard solutions in the linear range (10–1000 ng/mL) are presented in Figure 4A. The corresponding calibration curve is shown in Figure 4B, which presents the current intensity vs. the decimal logarithm of the β-PV concentration, following the equation I (µA) = (0.41 ± 0.02) log [β-PV] (ng/mL) + (0.46 ± 0.05), r = 0.997. The limit of detection (LOD = 1.8 ng/mL) and limit of quantification (LOQ = 7.1 ng/mL) were determined through statistical regression analysis, using the equations LOD = 3.3 × S_y/x/_b and LOQ = 10 × S_y/x/_b (in which b is the slope and S_y/x_ is the standard deviation of the linear regression). The precision of the results was confirmed through repeatability and reproducibility studies, with coefficients of variation of 3.6% and 8.9%, respectively.

Interference studies were performed with other food allergenic proteins, such as Tropomyosin (TPM, an allergen commonly present in seafood); and Ara h 1 (a peanut allergen) and Api g 1 (a celery allergen), which do not derive from fish. These were selected due to their possible cross-contamination in packaged foodstuff and other samples that may contain distinct ingredients, other than seafood products. Additionally, Tetrodotoxin (TTX), an emerging marine toxin, was also included in the study. β-PV solutions (1000 ng/mL) containing 1000 ng/mL of each allergen or toxin were analyzed. In Figure 4C, the similarity (in %) of the signals of the Ara h 1-, Api g 1-, and TTX-containing solutions and a β-PV standard solution with the same concentration are presented. The analytical responses obtained were very similar: 96%, 101%, and 100%, respectively. However, for TPM, it was 139%, indicating that this allergen interferes in the analysis, which may be related to cross-reactivity.

Additionally, due to the absence of a certified reference material, to further assess the accuracy of the magnetic immunoassay’s results, increasing concentrations of β-PV (25, 50, 100, 250, 500, and 1000 ng/mL) were added to a cooked white rice extract (negative control, absence of β-PV, diluted 100× in B1), obtaining a linear range between the current intensity and log [β-PV] (I (µA) = 0.41 log [β-PV] (ng/mL) + 0.50). Figure 4D compares the results obtained for the analysis of β-PV in B1 and the food extract, demonstrating that no significant matrix effects were observed since the obtained slope of the calibration curves were similar (slope buffer/slope matrix = 0.98).

Furthermore, a positive sample (fresh salmon) extract was diluted (300× in B1) and spiked with β-PV (100 ng/mL), obtaining a recovery of 113% (CV = 6%). These results indicate that there was no interference from the sample matrix, further validating the accuracy of the assay’s results.

The stability of the NH-MBs modified with CAb (stored at 4 °C) was tested over the course of several weeks (Figure 4E); the CAb maintained its chemical integrity for at least 43 days, and, over this period, the assay’s response exhibited a CV of 19%.

### 3.4. Application of the Magnetic Immunoassay to Food Analysis

To assess the assay’s potential to detect and quantify β-PV in foods, several commercial foods were selected: samples containing food allergens other than β-PV (egg, celery, and peanut); samples containing β-PV included fresh (salmon and squid) and processed products (sardine pâté, vacuum-packed fish surimi, canned fish surimi, canned tuna, tuna pâté, tuna hamburger, and tuna from vacuum-packed pasta); and β-PV-free samples or those potentially containing β-PV (rice, soy lecithin, liquid protein yoghurt, broccoli and cauliflower soup, roast chicken, quinoa salad, and seafood shrimp patty). These samples were chosen to evaluate the assay’s response in the presence of proteins from different plant and animal sources. The fresh and processed products containing β-PV were included to assess the impact of food processing on its allergen stability.

**Figure 5:**
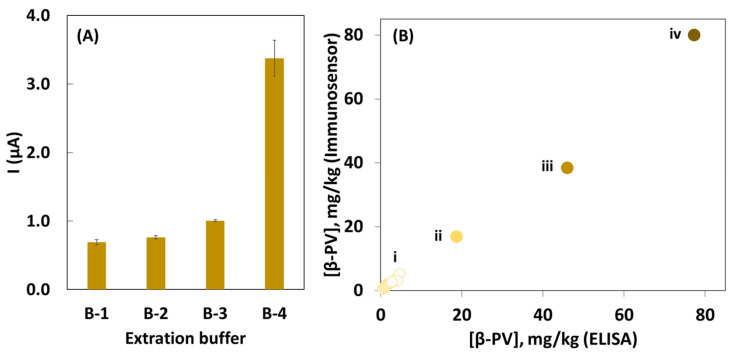
(**A**) Effect of β-PV extraction buffer on the signal using a fresh salmon sample: B-1—10 mM PBS with 5 mM EDTA, pH 7.4; B-2—10 mM PBS with 137 mM NaCl, pH 7.4; B-3—100 mM Tris buffer, pH 7.4, and B-4—16 mM Na_2_CO_3_ with 128 mM NaCl, pH 9.6. (**B**) Comparison of the results obtained by the immunoassay and the conventional ELISA for the following foods: i—egg, ground celery, peanut butter, rice, soy lecithin, liquid protein yoghurt, broccoli and cauliflower soup, roast chicken, sardine pâté, canned fish surimi, squid, canned tuna, and tuna burger; ii—fish surimi; iii—fresh salmon; and iv—tuna pâté.

To select the extraction buffer, a fresh salmon sample was chosen, and four extraction buffers reported in the literature were tested: (1) 10 mM PBS with 5 mM EDTA, pH 7.4; (2) 10 mM PBS, containing 137 mM NaCl, pH 7.4; (3) 100 mM Tris buffer, pH 7.4; and (4) 16 mM Na_2_CO_3_ containing 128 mM NaCl, pH 9.6 [21]. As shown in Figure 5A, buffer 4 was demonstrated to be the most efficient for β-PV extraction, as the obtained electrochemical signal was between 3.4 and 4.9 times higher than for the other buffers. This indicates that the pH of the buffer is fundamental for the extraction of this allergen, probably because of the degree of protonation of the protein. Table 1 summarizes the samples’ characteristics and the quantified β-PV amount (mg/kg). Furthermore, to validate the assay’s results, an ELISA was used to analyze the same samples. According to the results, the developed magnetic immunoassay demonstrates high specificity toward the fish allergen, as the concentration of β-PV obtained for the analysis of samples without β-PV was below the LOD of the method. Regarding the samples containing β-PV, the electrochemical sensor proved to be effective in quantifying the allergen. The samples with the highest β-PV quantities were vacuum-packed pasta tuna, tuna pâté, and fresh salmon (664.0, 80.0, and 38.6 mg/kg, respectively). Another interesting detail lies in the fact that food processing may affect the detection and quantification of β-PV. The processing/conservation methods of fish surimi (vacuum-processed and preserved in oil) yielded a β-PV quantity of 16.9 mg/kg and the LOQ value, respectively. In addition, sardine pâté, canned fish surimi, squid, canned tuna, and tuna burger had an allergen concentration above the LOD value, indicating the presence of β-PV, but below the LOQ. The low quantity of β-PV in samples such as sardine pâté, canned tuna or tuna burger can be explained by the processing method (heating the food between 75 and 100 °C for a relatively long time) because this leads to the denaturation of the protein, and antibodies do not recognize the allergen when it loses its structural integrity.

The assay’s results were compared with the ones obtained by a conventional ELISA, and an excellent correlation was obtained (Figure 5B), confirming their accuracy. It is also observed that different levels of β-PV can be identified according to the sample type: I—samples with [β-PV] below the analytical thresholds (egg, ground celery, peanut butter, rice, soy lecithin, liquid protein yoghurt, vegetable (broccoli and cauliflower) soup, roast chicken, sardine pâté, canned fish surimi, squid, canned tuna, and tuna burger); II—vacuum-packed fish surimi sample; III—fresh salmon sample; and and IV—tuna pâté. The samples are grouped according to the increasing quantity of the quantified allergen.

Another important observation is that the food processing and conservation affects the concentration of this allergen.

### 3.5. Comparison with Other Immunosensors and Immunoassays for PV Analysis in Food Samples

The analytical characteristics of the developed immunoassay were compared with recently reported immunosensors or immunoassays for the analysis of β-PV in foods (Table 2). In our previous work [22], a sandwich-type immunosensor using an SPCE modified with rGO-AuNP as its transducer was developed. Although this sensor demonstrated significant potential as an analytical tool for food quality control, a higher LOD (9.9 ng/mL) and a shorter stability were observed. Other studies, while presenting effective strategies for β-PV detection, showed significantly higher LODs (4.29, 65.0, and 10.15 ng/mL, respectively) [10,29,30]. Additionally, the stability of these sensors was not reported, thus making it difficult to assess their potential for long-term use or commercialization. An electrochemical sensor based on magnetic beads with fluorescent nanoparticles was reported, but the tested food samples were limited, with no negative controls to assess cross-reactivity [31]. Another study presented an ELISA, but, although an interesting analytical performance was achieved, no stability or interference studies were reported [32]. However, other studies exhibited lower LODs [32].

## 4. Conclusions

A magnetic immunoassay with electrochemical detection was developed to determine the fish allergen β-PV. Structural characterization of maleimide-functionalized magnetic beads by electron microscopy revealed spherical particles with a uniform size (1 µm) that are suitable to be used as a platform for biomodification with β-PV antibodies. The ability to retain the magnetic sandwich-type assay on the SPCE working electrode enabled efficient detection and quantification of the allergen through an enzymatic reaction, monitored by chronoamperometry. The analytical performance of the developed electrochemical immunosensor was studied, providing selective, precise, and accurate analysis of β-PV (CV of 3.6% and 8.9% for repeatability and reproducibility), and good recovery rates were achieved (113%, in fresh salmon). Potential interferents were analyzed (TPM, Ara h 1, Api g 1, and TTX), and the biosensor proved to be specific for β-PV. Nineteen commercial food samples were tested to assess the potential of this sensor for practical applications in food quality control containing proteins from different sources (animal or plant) and distinct culinary processing/conservation methods (fresh, grilled, fried, vacuum-packed, oil-preserved, or pre-cooked). The sensor detected β-PV in samples containing fish in their nutritional composition, and the highest β-PV contents were quantified in fish surimi (16.9 mg/kg), fresh salmon (38.6 mg/kg), tuna pâté (80.0 ng/mL), and vacuum-packed pasta tuna (664 mg/kg). The β-PV quantifications using the immunoassay were correlated with the conventional ELISA method, highlighting the potential of this analytical tool for quality control in the seafood industry. Furthermore, because of the assay’s high selectivity, it can also be employed in other food industries to detect cross-contamination that may occur during food processing and culinary practices, on working surfaces, etc., ensuring food safety.

## Figures and Tables

**Table 1:** β-PV amount (mg/kg) and respective coefficient of variation (CV, %) in commercially available foods, determined by the developed amperometric immunoassay and a conventional ELISA. (Samples were diluted 100×, except for the fresh salmon sample (300×) and the tuna pâté sample (400×).)

		Sample	Protein Origin	Fish-Derived Species (%)	Food Processing /Conservation	Immunoassay		ELISA	
						[β-PV] (mg/kg)	CV (%)	[β-PV] (mg/kg)	CV (%)
**β-PV-free**	Allergen	Egg	Animal	n/a	Ground	<LOD	–	<LOD	–
		Celery	Vegetable						
		Peanut butter	Vegetable						
	Other	Rice	Vegetable	n/a	Cooked	<LOD	–	<LOD	–
		Soy lecithin	Vegetable		Ground				
		Liquid protein yoghurt	Animal		Pasteurized				
		Vegetable soup	Vegetable		Lyophilized				
		Roast chicken	Animal		Grilled				
**Presence of β-PV**	Contain	Salmon (fresh)	Animal	100	n/a	38.6	15	46.0	24
		Sardine pâté		58	Pâté	<LOQ	-	<LOQ	–
		Canned fish Surimi		100	Canned in oil	<LOQ	-	<LOQ	–
		Fish surimi		100	Packed in vacuum	16.9	22	18.7	27
		Squid (fresh)		100	n/a	<LOQ	-	<LOQ	–
		Canned tuna		100	Canned	<LOQ	-	<LOQ	–
		Tuna pâté		45	Pâté	80.0	30	77.3	22
		Tuna burger		52	Packed in vacuum	<LOQ	-	<LOQ	–
		Pasta with tuna		40	Packed in vacuum	664	9	<LOQ	–
	May contain	Quinoa salad	Vegetable	n/a	Packed in vacuum	<LOD	–	<LOQ	–
		Seafood shrimp patty	Animal	30	Fried				

**Table 2:** Summary of analytical characteristics of electrochemical immunosensors and/or ELISA.

Sensing Surface/Platform	Stability (Days)	Technique	Probe/Label	LOD (ng/mL)	Food Samples	Interferents Tested	Ref.
SPCE/NH-MBs	43	CA	HRP/TMB	1.8	Egg, ground celery, peanut butter, rice, soy lecithin, liquid protein yoghurt, vegetable soup, roast chicken, salmon (fresh), sardine pâté, canned fish surimi, fish surimi, squid (fresh), canned tuna, tuna pâté, tuna burger, pasta with tuna, quinoa salad, seafood shrimp patty	TPM Ara h 1 Api g 1 TTX	This work
SPCE/rGO-AuNP	15	CA	HRP/TMB	9.9	Salmon (fresh, grilled, canned, smoked, pâté), cod (smoked, canned), tuna (canned), sardines (canned), needlefish (canned), mackerel (canned), broth (fish, seafood, and stew), vegetable soup, lettuce, and egg	TPM Ara h 1 Gal d 3 Cyp c 1 TTX	[22]
MGCE/CMFNPs	–	EIS	[Fe(CN)_6_]^3-/4-^	0.16	Crucian carp and brown shrimp	Pen a 1 β-conglycinin Ara h 1	[31]
Microtiter plate/MB-GO	–	ELISA	HRP-Ab-AuNP	4.29	Fish (*Ctenopharyngodon idellus, Aristichthys nobilis, Oreochroms mossambcus,* and *Gadus macrocephalus*)	BSA OVA, TPM, AK, COL	[30]
Microtiter plate	–	ELISA	HRP/TMB	0.20	Aquatic products, cereal products, baby food, snacks, dips and sauces, beverages, and other edible products	–	[32]
Graphite rod electrode/Au-Cu-MOF-PDA-MWCNT	–	DPV	Endothelial cells/NO	65.0	Carp	BSA, OVA, Soybean Globulin COL	[29]
Microtiter plate	–	ELISA	HRP/TMB	10.15	Seafood-flavored puffed potato, cod stick, crab-flavored fish stick, Antarctic krill ball, grilled fish fillet, seafood noodle, bear-shaped biscuit, hand-torn bread, ice cream, chocolate, crab-flavored broad bean, and cheese sticks	–	[10]

Ab—antibody; AK—arginine kinase; Au-Cu-MOF-PDA-MWCNT—gold–copper–metal–organic framework polydopamine-modified multi-wall carbon nanotubes; AuNP—gold nanoparticles; BSA–bovine serum albumin; CA—chronoamperometry; CMFNPs—cationic magnetic fluorescent nanoparticles; COL—collagen; DPV—differential pulse voltammetry; ELISA—enzyme-linked immunosorbent assay; EIS—electrochemical impedance spectroscopy; HRP—horseradish peroxidase; LOD—limit of detection; MB-GO—magnetic beads–graphene oxide; MGCE—magnetic glassy carbon electrode; NH-MBs—maleimide-functionalized magnetic beads; NO—nitric oxide; rGO—reduced graphene oxide; SPCE—screen-printed carbon electrode; TMB—3,3′,5,5′-Tetramethylbenzidine; TPM—tropomyosin; TTX—tetrodotoxin.

## Data Availability

Data are contained within the article.

## References

[1] Permyakov E.A., Uversky V.N. (2022). What Is Parvalbumin for?. Biomolecules.

[2] Lee P.-W., Nordlee J.A., Koppelman S.J., Baumert J.L., Taylor S.L. (2012). Measuring parvalbumin levels in fish muscle tissue: Relevance of muscle locations and storage conditions. Food Chem..

[3] Saidi A., Cavallo C., Del Giudice T., Vecchio R., Cicia G. (2023). Consumer preferences for finfish: A systematic literature review. Food Qual. Prefer..

[4] Mukherjee S., Horka P., Zdenkova K., Cermakova E. (2023). Parvalbumin: A Major Fish Allergen and a Forensically Relevant Marker. Genes.

[5] Sun Y., Luo Y., Chen J., Liu X., Gao J., Xie Y., Chen H. (2024). Fish Allergy: A Review of Clinical Characteristics, Mechanism, Allergens, Epitopes, and Cross-Reactivity. ACS Food Sci. Technol..

[6] FAO, WHO (2022). Risk Assessment of Food Allergens: Part 2: Review and Establish Threshold Levels in Foods for the Priority Allergens: Meeting Report.

[7] Sun L., Lin H., Li Z., Sun W., Wang J., Wu H., Ge M., Ahmed I., Pavase T.R. (2019). Development of a method for the quantification of fish major allergen parvalbumin in food matrix via liquid chromatography-tandem mass spectrometry with multiple reaction monitoring. Food Chem..

[8] Sebastián-González E., Morales-Reyes Z., Botella F., Naves-Alegre L., Pérez-García J.M., Mateo-Tomás P., Olea P.P., Moleón M., Barbosa J.M., Hiraldo F. (2020). Network structure of vertebrate scavenger assemblages at the global scale: Drivers and ecosystem functioning implications. Ecography.

[9] Dasanayaka B.P., Li Z., Ahmed A.M.M., Zhao J., Lin H. (2024). Different Antibody Reactivity and Its Implications in Allergen Detection in Processed Fish. ACS Food Sci. Technol..

[10] Huang Y., Zhu W., Wu Y., Sun L., Li Q., Pramod S.N., Wang H., Zhang Z., Lin H., Li Z. (2024). Development of an indirect competitive ELISA based on the common epitope of fish parvalbumin for its detection. Food Chem..

[11] Dhruve D., Ratrey V.P., Lowanshi A., Chandravanshi S., Kashyap N., Pandey S. (2024). Comprehensive Review of Seafood Allergens: Regulations, Detection Methods, and Implications for Public Health. J. Aquat. Food Prod. Technol..

[12] Fu L., Wang C., Zhu Y., Wang Y. (2019). Seafood allergy: Occurrence, mechanisms and measures. Trends Food Sci. Technol..

[13] Lu Y., Ohshima T., Ushio H. (2004). Rapid Detection of Fish Major Allergen Parvalbumin by Surface Plasmon Resonance Biosensor. J. Food Sci..

[14] Shin J.H., Reddy Y.V.M., Park T.J., Park J.P. (2022). Recent advances in analytical strategies and microsystems for food allergen detection. Food Chem..

[15] Xu J., Ye Y., Ji J., Sun J., Sun X. (2022). Advances on the rapid and multiplex detection methods of food allergens. Crit. Rev. Food Sci. Nutr..

[16] Curulli A. (2022). Recent Advances in Electrochemical Sensing Strategies for Food Allergen Detection. Biosensors.

[17] Freitas M., Carvalho A., Nouws H.P.A., Delerue-Matos C. (2022). Tracking *Arachis hypogaea* Allergen in Pre-Packaged Foodstuff: A Nanodiamond-Based Electrochemical Biosensing Approach. Biosensors.

[18] Freitas M., del Rio M., Nouws H.P.A., Delerue-Matos C. (2022). Tracking a Major Egg Allergen to Assess Commercial Food Label Compliance: Towards a Simple and Fast Immunosensing Device. Biosensors.

[19] Sheng K., Jiang H., Fang Y., Wang L., Jiang D. (2022). Emerging electrochemical biosensing approaches for detection of allergen in food samples: A review. Trends Food Sci. Technol..

[20] Teixeira J.S., Freitas M., Oliveira C., Pereira C.R., Delerue-Matos C., Nouws H.P.A. (2024). Voltammetric immunosensor based on oxidized carbon nanotubes/MnFe_2_O_4_ hybrid nanoplatform for amplified detection of celery (*Apium graveolens*). Food Chem..

[21] Torre R., Freitas M., Costa-Rama E., Nouws H.P.A., Delerue-Matos C. (2022). Food allergen control: Tropomyosin analysis through electrochemical immunosensing. Food Chem..

[22] Rocha J.P., Freitas M., Geraldo D., Delerue-Matos C., Nouws H.P.A. (2024). Seafood product safety: A hybrid graphene/gold-based electrochemical immunosensor for fish allergen analysis. Food Chem..

[23] Fortunati S., Giannetto M., Giliberti C., Mattarozzi M., Bertucci A., Careri M. (2024). Magnetic Beads as Versatile Tools for Electrochemical Biosensing Platforms in Point-of-Care Testing. Anal. Sens..

[24] Gaiani G., O’Sullivan C.K., Campàs M. (2019). Magnetic Beads in Marine Toxin Detection: A Review. Magnetochemistry.

[25] Piroozmand F., Mohammadipanah F., Faridbod F. (2020). Emerging biosensors in detection of natural products. Synth. Syst. Biotechnol..

[26] Liu D., Zhou Y., Gao S., Tang Z., La M. (2022). Overview on the design and application of magnetically-assisted electrochemiluminescence biosensors. Int. J. Electrochem. Sci..

[27] Cheng H.-P., Yang T.-H., Wang J.-C., Chuang H.-S. (2024). Recent Trends and Innovations in Bead-Based Biosensors for Cancer Detection. Sensors.

[28] Carinelli S., Luis-Sunga M., González-Mora J.L., Salazar-Carballo P.A. (2023). Synthesis and Modification of Magnetic Nanoparticles for Biosensing and Bioassay Applications: A Review. Chemosensors.

[29] Jiang D., Feng Z., Jiang H., Cao H., Xiang X., Wang L. (2024). 3D bio-printing-based vascular-microtissue electrochemical biosensor for fish parvalbumin detection. Food Chem..

[30] Wang Y., Li H., Zhou J., Qi Q., Fu L. (2020). A colorimetric and fluorescent gold nanoparticle-based dual-mode aptasensor for parvalbumin detection. Microchem. J..

[31] Jiang D., Zhu P., Jiang H., Ji J., Sun X., Gu W., Zhang G. (2015). Fluorescent magnetic bead-based mast cell biosensor for electrochemical detection of allergens in foodstuffs. Biosens. Bioelectron..

[32] Wu Y., Lin H., Lu Y., Huang Y., Dasanayaka B.P., Ahmed I., Chen G., Chen Y., Li Z. (2021). Allergenicity determination of *Turbot parvalbumin* for safety of fish allergy via dendritic cells, RBL-2H3 cell and mouse model. Eur. Food Res. Technol..

